# Management of Failed External Fixation by Two‐Staged Internal Osteosynthesis in the Lower Limb

**DOI:** 10.1111/os.12870

**Published:** 2021-01-19

**Authors:** Bahaa Ali Kornah, Mohamed Abdelaziz, Mohamed I Abulsoud, Tharwat Abdel Ghani, Nagi Seleem, Ehab A. Alshal, Mohamed Aly Abdel‐AAl

**Affiliations:** ^1^ Department of Orthopedics Faculty of Medicine, AL‐Azhar University Cairo Egypt; ^2^ Elbakry General Hospital Ministry of Health Cairo Egypt

**Keywords:** External fixation, Locked compression plate, Lower limb trauma, two‐stage reconstruction, failed external fixation, Non‐union, Open fractures

## Abstract

**Objective:**

This study aims to evaluate the result of a two‐stage (delayed conversion) management of nonunion after failed external fixation of the lower limb.

**Methods:**

A case series of 25 patients (19 males and six females) enrolled in this study between February 2008 and October 2016, mean age 33.4 years (range, 22–65 years). Eight had femoral fractures, and 17 had tibial fractures. All were due to high‐energy trauma and were open fractures. All cases were presented by non‐union after external fixation in the lower limb long bones. All patients were managed by two stages (delayed conversion) osteosynthesis. The patients have been assessed for rate and time for union, range of motion of adjacent joints, the Modified functional outcome score of Karlstrom‐Olerud, and Trauma outcomes measure score.

**Results:**

The mean follow‐up was 36.5 months (range 24–54 months). Twenty‐two cases (88%) were fully united on an average of 5.3 months. According to the Karlstrom‐Olerud scores, the final functional outcome score was excellent 12 cases, good 9 cases, accepted 2 cases, and poor in two cases. As regards the trauma outcome measure score, the mean TOM after 3 months was 26.1 (25.3–27.3), 30.4 (29.3–32.1) after 12 months, and 33.4 (32.3–40) after 24 months.

**Conclusions:**

The technique of two‐stage treatment of nonunions of long bone after external fixation is a successful tool to achieve bony union. It could be a favorable option with a low risk of complications and a high level of functional outcomes.

## Graphical abstract

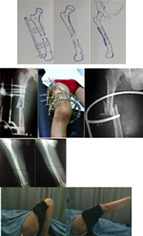

In cases of failed external fixation of the lower limb, The technique of two stage internal fixation is highly successful with 88% union rate and 84% satisfactory functional outcome.

## Introduction

One of the most common indications for external fixation in the management of fractures associated with high injury trauma which is commonly comminuted and open ones.^1^ There were still many questions and problems with its use. Furthermore, the optimal frame design and biomechanical characteristics of each fixator still controversial^2^.

The incidence of non‐union following high injury trauma ranges from 5% to 20.3%.^3^, ^4^, ^5^ The causes of non‐union include soft tissue damage, high injury trauma, improper reduction, the frame used in external fixation, supplemental use of an interfragmentary screw, postoperative infection, and dynamization at the fracture site. The nonunion is almost an atrophic type according to Weber–Cech classification^6^. Despite distraction osteogenesis in limb reconstruction, surgery is a good option, prolonged external fixation is troublesome for the patient and associated with complications^7^, ^8^, ^9^, ^10^, ^11^. The risk of combining external and internal fixation is deep infection. The reported incidence is 3%–15%^12^, ^13^. There is no consensus about the method of internal fixation when used after external fixation, which leads to optimal results. There are two methods: acute conversion and delayed conversion^14^. The acute conversion consists of removal of the external fixator device and performing internal fixation in the same setting. The delayed conversion consists of two separate procedures for the removal of external fixation and the insertion of the internal fixation device.

The protocol for the treatment is two‐stage management (delayed conversion); the first surgery includes removal of the fixator and Debridement of external fixation pin tracks. Stabilization of fractures has been individualized according to the site and stability by traction, plaster of Paris, and functional braces. This interval varied and the second procedure was performed when the pin tracks healed with no sign of infection. The second stage included the stabilization of fracture and bone grafting to enhance bone healing and halt nonunion^14^, ^15^, ^16^. This interval varied and the second procedure was performed when infection parameters were negative and pin‐tracks site healed with no infection.

The aim of this study was to assess the functional outcomes of the two‐stages (delayed conversion) management of nonunion after the failure of external fixation with the purpose of achieving bone healing and preserving the range of motion of the joints of the lower limb.

## Patients and Methods

This is a case series included 25 patients (19 males and 6 females), the study population is adult patients who underwent two‐stage conversion to internal fixation between 2008 and 2016. All the patients had un‐united fractures in long bones of the lower limb after failed external fixation were enrolled in this study. Exclusion criteria included age below 18 years old, patients with major neurovascular compromise, known cases of peripheral vascular disease, complex deformities, and patients who were treated primarily by a different technique than external fixator.

The mean follow‐up was 36.5 months (range 24–54 months). Mean age 33.4 years (range, 22–65 years). Eight had femoral fractures and 17 with tibial fractures. All were due to high‐energy trauma and were open fractures. Fracture types are categorized according to AO classification. There were nine cases with type (A), eight cases were type (B), and eight cases were typed (C), and according to Gustillo‐Anderson classification, three cases were grade (I), 15 cases were grade (II), and seven cases were grade (III). In addition, there were four cases cases initially treated by external fixators owing to the presence of bone loss, nine cases of fracture with associated severe soft tissue injuries, and 12 cases of severely comminuted diaphyseal or metaphyseal fracture. The reasons for conversion to internal fixation were patients' incompliance with external fixation for 13 patients, persistent pin tract infections in 10 patients, docking site‐related problems in 2 patients. All cases show clinical and radiological signs of non‐union.

The demographic distribution of the cases was as follows. The Mean primary surgeries had been done per case was 2.3 (range one to four operations). It included serial debridement, revisions, or realignment of the fixator and bone grafting. five cases were presented at the time of presentation by active infection and one case has deep sequestrum.

Preoperative assessment included history involving General health of the host and presence of medical co‐morbidities, social habits (smoking and alcohol consumption), and previously offered treatment for the fracture, complications, time of referral, occupation. Soft tissue envelope, Joint contracture, range of motion, Nerve function, Vascular status, Location and type of nonunion, and Psychosocial resources.

Evaluation for signs of infection involved complete blood cell count (CBC), erythrocyte sedimentation rate (ESR), C‐reactive protein (CRP) for all cases. Also, the patients' nutritional status was assessed by total lymphocytic count and Rainey‐MacDonald nutritional index.

Imaging studies included standard anteroposterior, lateral, and oblique views to assess: the degree of bone gapping, evaluation of medullary cavity, and presence of sequestrum or signs of infection and deformity and its plane. Surgery planning included the use of radiographs of the uninvolved side with an overlay on tracing paper to determine the bony alignment, length, type, and design of the implant to be used.

Ethical approval was obtained from the institutional ethics committee and informed consent forms from all patients were received.

## Surgical Procedure

**Fig 1:**
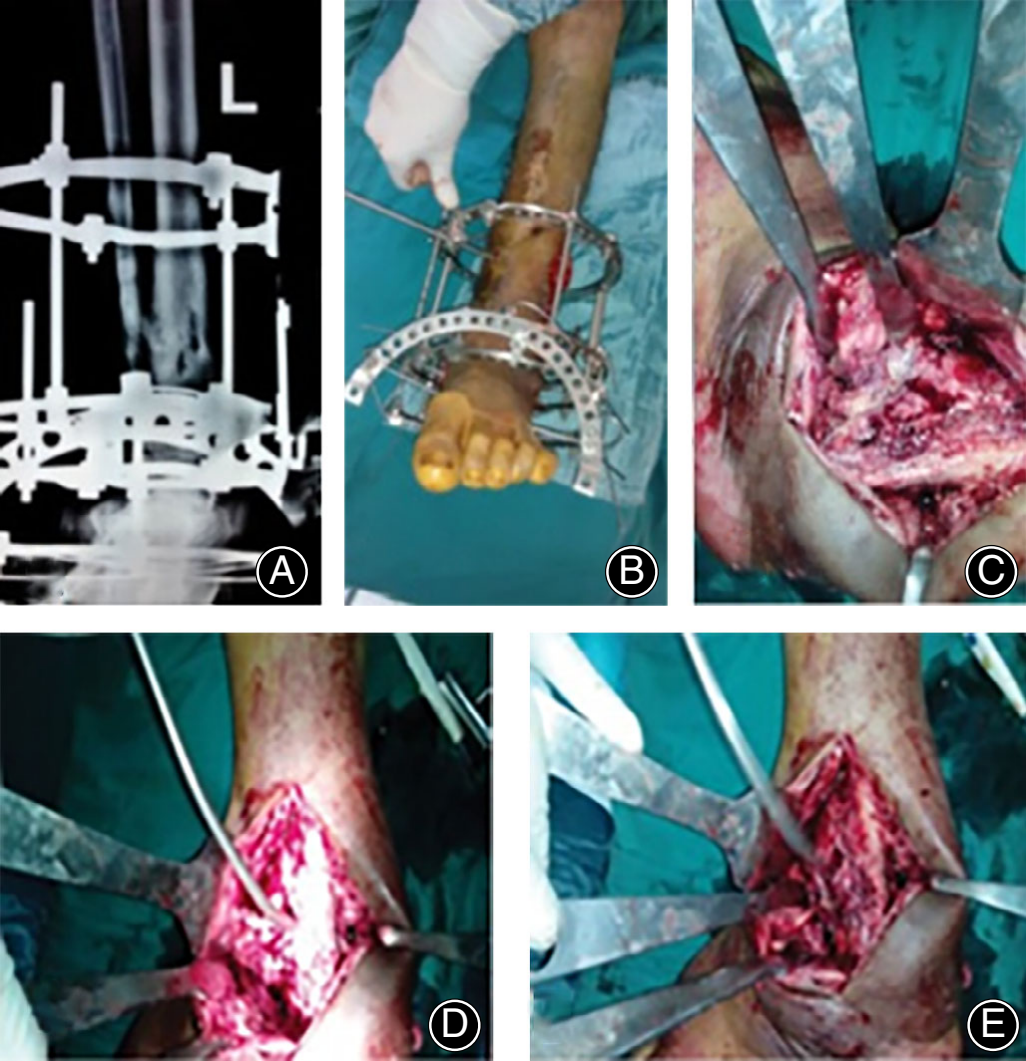
Operative details of non‐united lower 3rd tibia. (A) Fracture nonunion in Ilizarov fixator (B) Photo of the frame (C) Intraoperative showing site of nonunion (D and E) Debridement of bone and pin track.

All cases have been performed under regional (spinal or epidural) anesthesia. In the first stage, removal of the external fixator and debridement of pin track was been performed for all cases. For infected cases, soft tissue was thoroughly debrided and the site of pseudoarthrosis was identified and all soft tissues' interposition removed with sequestrectomy and aggressive debridement of all necrotic tissues. Tissue samples have been obtained from multiple sites for cultures and sensitivity. Surgical wound lavage is performed using normal saline with an antiseptic (Povidone‐iodine 7.5% solution) for removing blood clots and devitalized tissues (Fig. 1). Wound closure was performed in 19 cases, leaving a suction drain for removal within 48 hours after surgery. Two cases required secondary skin graft for wound closure. The remaining cases were treated by removal of external fixation and pin tract debridement. The limb was stabilized by plaster of Paris cast (nine cases), traction (eight cases), functional braces (five cases), and removable splint (three cases). Monotherapy broad‐spectrum antibiotics started (3rd generation cephalosporin) until the results of the culture appear. Patients were discharged with regular outpatient clinic attendance and monitored weekly until the infection parameters became within normal limits.

**Fig 2:**
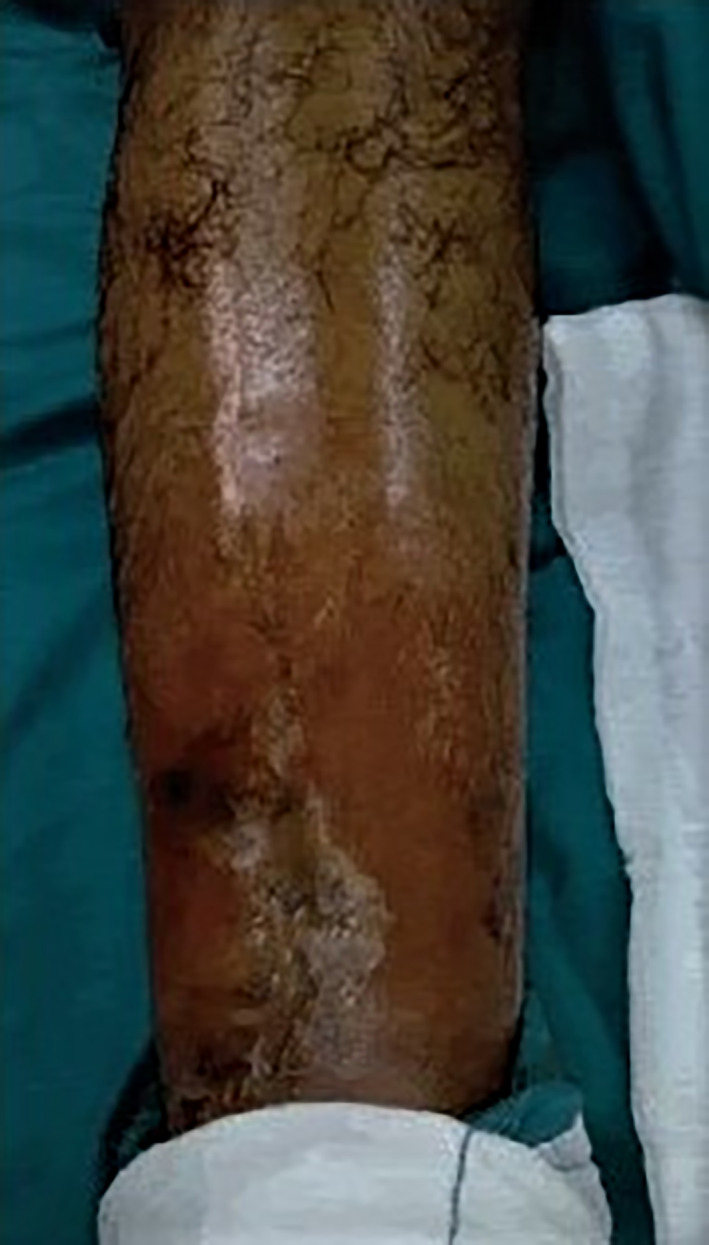
(A) Adult patient treated by ring fixator with no evidence of union after 10 months, (B) X‐ray show nonunion of the fracture and removal of fixator, (C) and (D) Post‐operative X‐ray shows plate fixation and bone grafting.

In the second stage, after all clinical and laboratory signs of infections have been resolved, the patient was readmitted. During surgery, the nonunion site was re‐exposed and re‐evaluated for any residual infection. Excessive release of the soft tissues attached to bone has been avoided to preserve their blood supply. Trimming and shingling of bony ends were carried out (together with the opening of the medullary canal) to get maximum bone contact and achieve an inherently stable fracture. In some cases, an AO/ASIF femoral distractor is applied and used to correct the length and alignment of bone without marked soft tissue stripping and devitalization. Internal fixation is undertaken. The choice of the fixation depended upon the level of the fracture, presence of an open medulla, and preferences of the individual surgeons. Locked plates (owing to the co‐existence of osteoporosis) was used in 18 patients, locked broad dynamic compression plate in two cases, locked narrow compression plate in four cases, locked distal tibial plate in five cases, locked proximal tibial plate in six cases, while Interlocking femoral nail was used in seven cases, and locked distal femoral plate in one case. The length of the plate was chosen so that the plate span ratio is about 3 and the plate screw density ranges from 0.5 to 0.6. Five patients needed manipulation to improve joint range of motion. Following stable fixation, autogenous bone grafts are applied around the nonunion to fill the gap if present and to accelerate bone healing. Bone grafts were harvested from the iliac crest (23 cases) and in two cases additional ipsilateral non‐vascularized fibular graft was needed. Wound was then closed over a suction drain. All cases were operated by the authors with the same technique (Fig. 2).

### *Postoperative Management*

Patients were instructed for toe‐touch weight‐bearing with crutches from the first day postoperatively and for 3 weeks. Progressive increase in weight‐bearing was encouraged. Full weight‐bearing was allowed when radiological signs of bone healing appear (2–5.5 months) with a mean of 4.2 months. Rehabilitation protocol started from the second postoperative day with isometric and active assisted exercises of all joints to improve the stiffness of the joints developed during the period of the fixators. Antibiotics were given for 1 week. Patients have been reviewed clinically on a regular basis after 2 weeks, 6 weeks, 3 months, 6 months, and 1 year with serial radiographs. The functional results have been evaluated using trauma outcomes measure (TOM) which is proposed by the AO foundation^17^. It shows the state of progression or regression of the functional outcomes during the follow‐up period. The questionnaire has been translated into the Arabic language and ensured that the patient perceived and understand it fully before filling the questionnaire.

At the final follow‐up visit, pain associated with weight‐bearing, any leg‐length discrepancy, limb alignment, and range of movement of hip, knee, and ankle have been evaluated.

Radiographic evaluation included: bone union, malunion, axis deviation, limb shortening, and any evidence of implant failure. The final functional outcome was evaluated according to the modified functional evaluation of the Karlstrom–Olerud score^18^.

#### *Statistical Analysis*

Data were analyzed using Statistical Program for Social Science (SPSS) version 15.0 (SPSS Inc., Chicago, Illinois). Quantitative data were expressed as mean ± standard deviation (SD) after confirmation of its normal distribution. Qualitative data were expressed as frequency and percentage. P‐value <0.05 was statistically significant.

## Results

### *Follow Up*

The mean follow‐up was 36.5 months (range 24–54 months). The duration of nonunion was 9.8 months (range 8–13 months). The average delay before osteosynthesis (until no signs of infection) was 3 weeks (range 2–4 weeks). The time varied depending upon the condition of pin tracts, timing of infection eradication, and general condition of the patient.

### *General Results*

Twenty‐two cases (88%) were united completely on an average of 5.3 months (range 4–8 months) and three cases exhibited persistent nonunion which had been overcome by re‐grafting and injection of bone marrow aspirate (BMA). Union was assessed both clinically and radiologically. All united cases were able to walk with minimal pain or without pain. No deformity of more than 10° in any plane was detected. Limb shortening was detected in four cases (16%), with an average of 3o mm.

**Fig 3:**
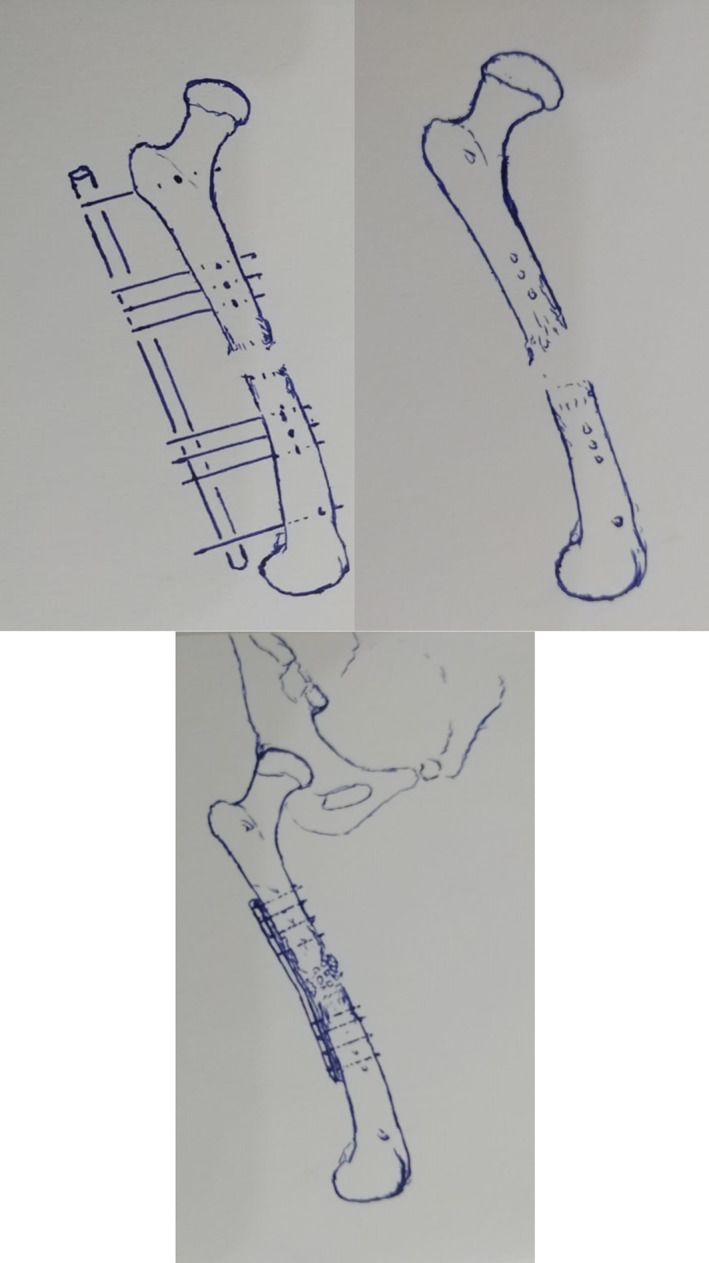
Clinical photo of a patient presented by deep infection with sequestrum.

All patients had a full range of motion at the hip and ankle joints. Eighteen (72%) regained full movement at the knee (compared to the other side) while seven patients (28%) had residual limited knee flexion varying from 15° to 30°. A superficial infection has been recorded in five cases (20%), and one case showed late infection with the formation of sequestrum in distal tibia and was operated upon and all responded well to antibiotic therapy (Fig. 3). No patient had implant failure nor residual knee instability.

As regards the trauma outcome measure score, the mean TOM after 3 months was 26.1 (25.3–27.3), 30.4 (29.3–32.1) after 12 months, and 33.4 (32.3–40) after 24 months (Table 1).

**TABLE 1:** Mean patient‐reported scores for the TOM

Outcomes	Number of patients	3 months	6 months	12 months
TOM	25	26.1 (25.3–27.3)	30.4 (29.3–32.1)	33.4 (32.3–40)

TOM, trauma outcomes measure.

According to the Karlstrom–Olerud scores, the final functional outcome score was excellent in 12 cases, good in nine cases, acceptable in two cases, and poor in two cases (Table 2).

**TABLE 2:** Distribution of cases according to the modified functional evaluation of the Karlstrom–Olerud score

Clinical results	No. of patients
Excellent	12
Good	9
Satisfactory	2
Moderate	‐
Poor	2

### *Complications*

Overall complications included: three cases of nonunion (12%), five cases of superficial infection (involving skin edges), and one deep infection (infection extended to muscles and fascia), seven cases of knee stiffness (residual limitation), and four cases of limb‐length discrepancy including one case of malunion.

**Fig 4:**
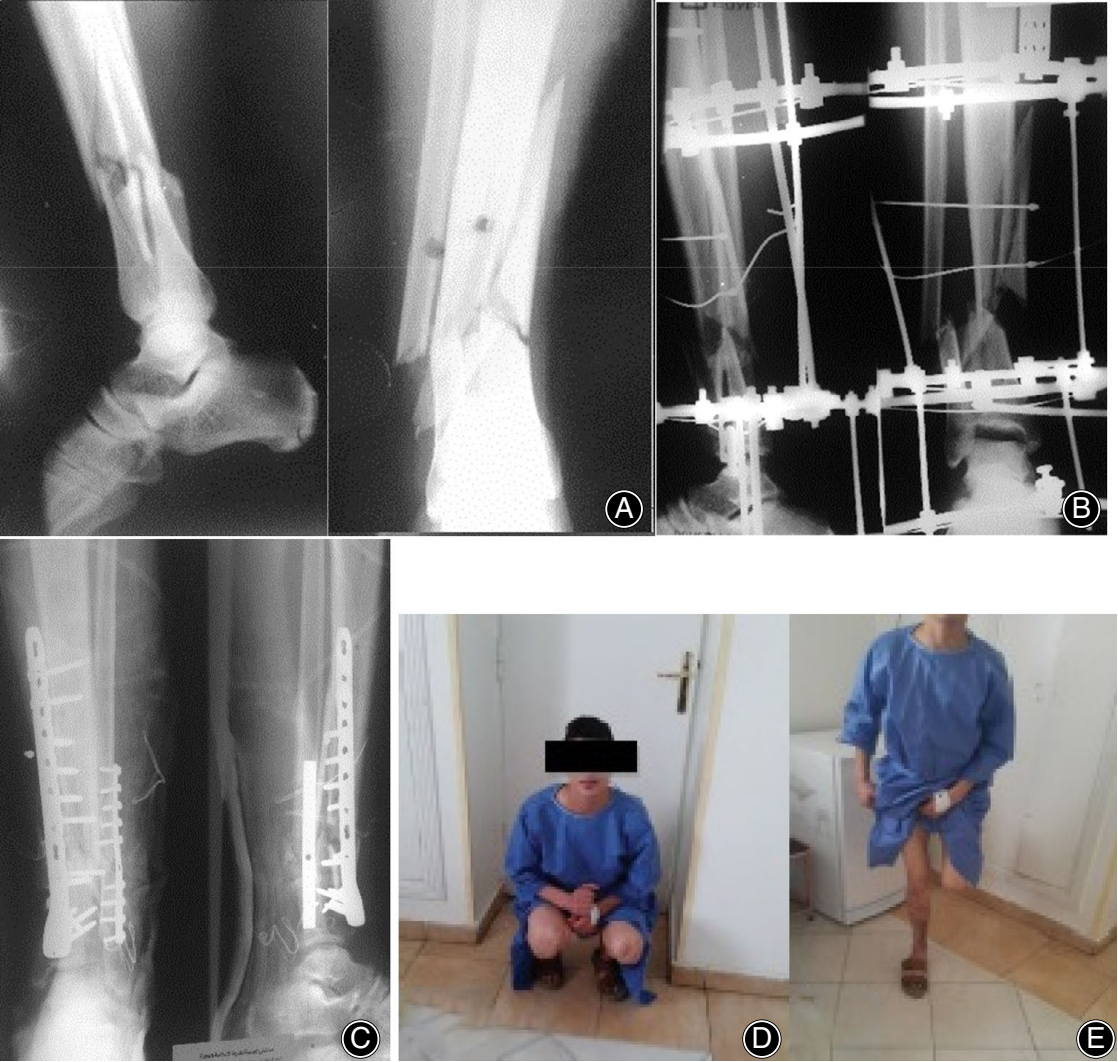
(A) 27‐year‐old male involved in road traffic accident treated initially by ring fixators, (B) X‐ray show ring fixators after 10 months with nonunion, (C) Internal fixation with distal tibial locked plate and bone grafting, (D) Excellent functional outcome (full knee flexion), (E) Full weight‐bearing on the affected limb.

**Fig 5:**
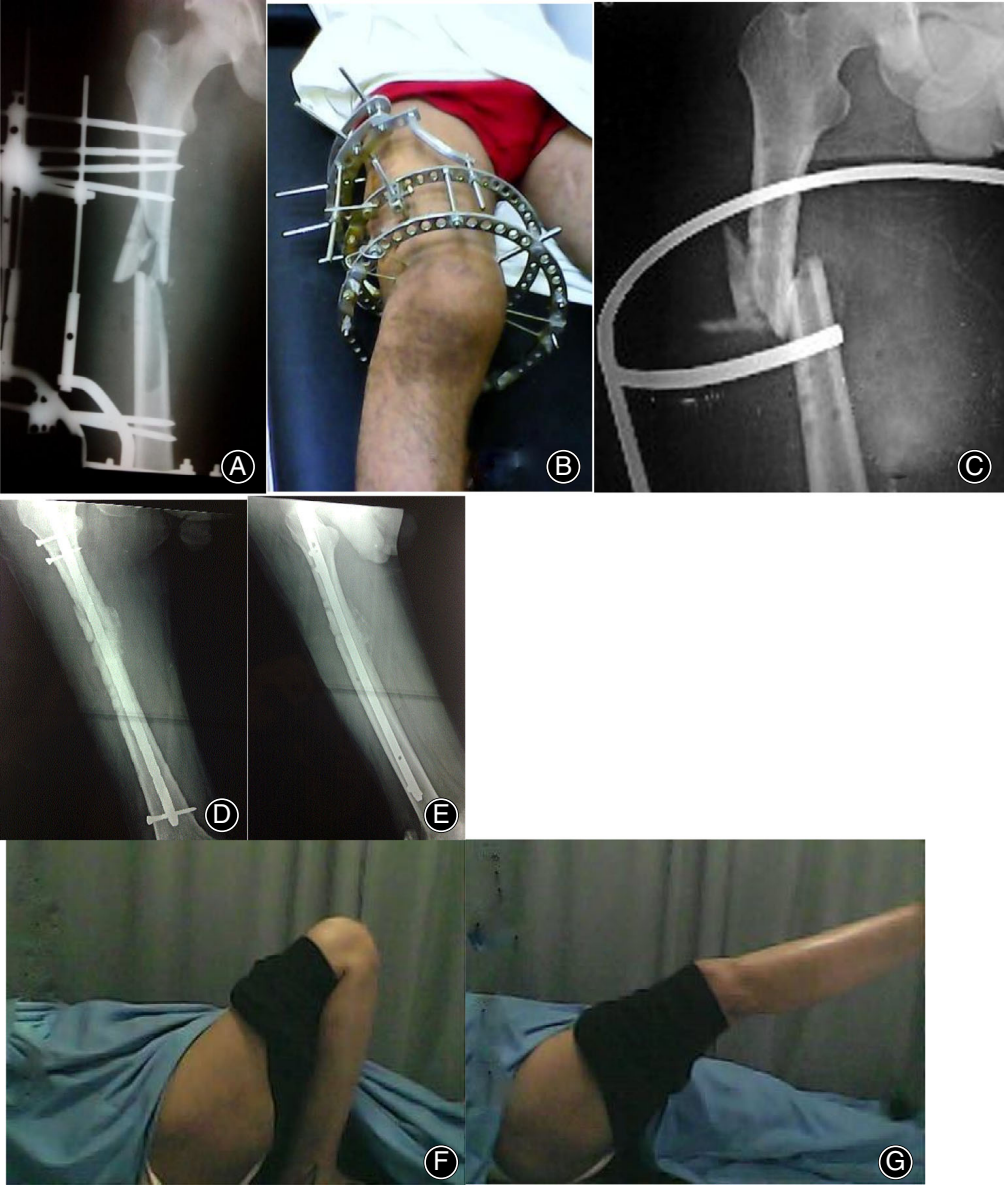
(A and B) Adult male with open comminuted shaft femur treated with ring fixator, (C) X‐ray of nonunion (after fixator removal) in splint during waiting until signs of infection resolve, (D) Immediate post‐operative X‐ray after fixation of the fractures by intramedullary interlocking nail and bone grafting, (E) Two months postoperative X‐ray with the healing of the fracture, (F) and (G) good range of motion of hip and knee joints.

**Fig 6:**
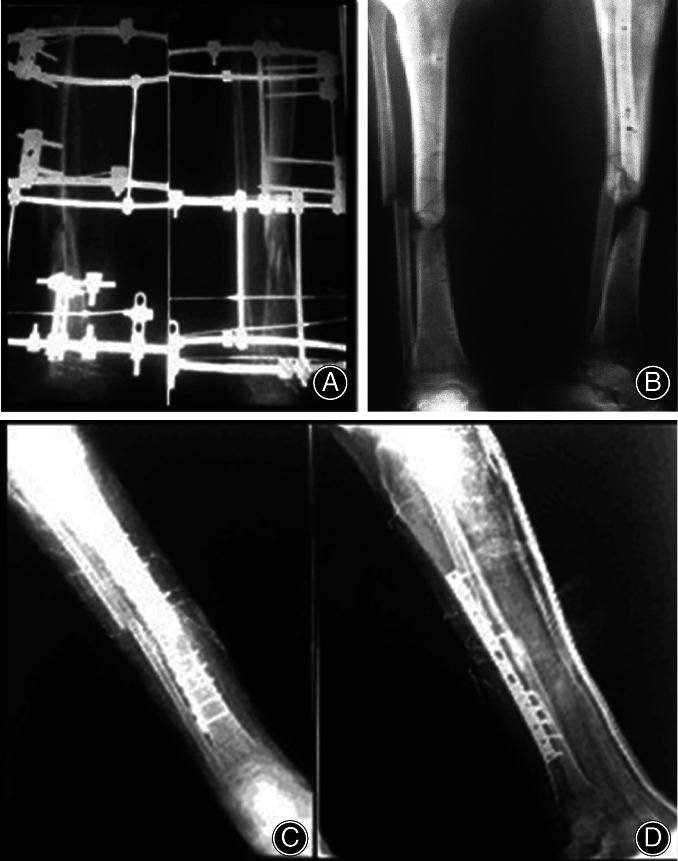
(A) Adult patient treated by ring fixator with no evidence of union after 10 months, (B) X‐ray show non‐union of the fracture and removal of fixator, (C and D) Post‐operative X‐ray shows plate fixation and bone grafting

Figures 4, 5 and 6 are case presentations.

## Discussion

Complex reconstruction surgery by distraction osteogenesis may lead to prolonged periods of external fixation. Due to the complexity of cases, a need for conversion to internal fixation owing to patient non‐compliance failed progression in treatment or persistent complications with continued use of the external fixator device^14^. It is not precisely proved when is the best time to convert to definitive fixation without increasing the risk of infection^19^.

Failure of fractures to unite after treatment with external fixation is a challenge for orthopaedic surgeons, particularly in cases of osteoporosis, joint stiffness, and persistent complications in addition to patient dissatisfaction. Nonunion could be due to the existence of a bone defect, persistent pin tract infection, mechanical instability, deficiency of biological environment essential to the bone to unite, and lack of experienced surgeon^20^. The qualities of the soft‐tissue envelope, blood supply around the fracture, mechanical stability at the fracture site are important factors for deciding the treatment modality^21^, ^22^. The management of this study aims to achieve sound union, early mobilization, and avoid complications. One of the common problems after external fixation application is the presence of pin tracks infection which renders the fractures potentially infected and liable to nonunion^12^, ^13^.

It was planned in this study to achieve our objective (healing of nonunion) through the strategy of (delayed conversion). This was to be achived by first converting the infected nonunion into non‐infected one *via* complete eradication of infection by thorough debridement of all involved soft tissue and removal of the fixator^14^. CRP is a helpful indicator of infection, but it is not necessarily specific and cultures are reliable tools though findings are often negative, especially in patients on antibiotics when cultures have been taken^15^. Also, the nutritional status of the patients has been assessed using the total lymphocyte count and nutritional index of Rainey‐MacDonald. both parameters can identify patients who are prone to develop infection postoperatively^16^. This prospective case series study provides some support for the strategy of conversion from external to internal fixation.

Many studies have focused specifically on nonunion after the failure of external fixation. Literature showed that achieving the bone union of non‐united fracture associated with open fractures managed by definitive external fixators is a great challenge when both intrinsic and extrinsic blood supply was violated by infection or multiple operations^17^.

We utilized locked compression plates (due to localized osteoporosis) which magnified the characteristics of biological fixation by the principle of internal fixators with more potential to preserve residual blood supply that was already affected by previous surgeries.

Comparing the results with other studies utilizes the same technique; Van den Bossche et al. reported on 57 patients mean union time of 40.6 weeks [21]. It is a considerably long time compared to the current study (5.2 months). This could be because most of our cases were grade (I) and grade II open fractures (18 cases), Also, proper debridement prevents the development of infection in addition to preserving the soft tissue attachment which avoids the occurrence of gapping and the use of bone grafting in all cases. Also, there was only one case of deep infection and one case of malunion. The functional outcome was good to excellent in all their cases. In this study, non‐union encountered in 3 cases and were operated by re‐grafting and bone marrow aspirate injection and they achieved union but still is relatively lower than many series. Wheelwright *et al*.^20^ showed that delay in secondary nailing until granulation of the pin sites is associated with a low infection rate. They reported a union rate of 11.6% and a 7% infection rate. Bernat *et al*.^20^ reported the outcome of 17 patients in whom the union rate was 100%, no deep infection, and two superficial ones. Monni *et al*.^21^ on dealing with five limbs for the delayed conversion of fixators to internal fixation reported 13.3% infection, and all their cases achieved full union.

The incidence of complications in the series is relatively low including knee stiffness (28%), deep infection (4%), and limb length discrepancy (16%). Infection remains a threat with prolonged external fixation and is a risk when the method of fixation is changed to internal fixation. it is recommended that pin tract infections should be treated first before the conversion is essential.

There were no cases of implant failure in this study, this could be attributed to the bone graft is used in all cases to overcome any bone defect and minimizes exposure of the plate to concentrated bending or torsional stresses which consequently predisposes to implant failure ^23^.

The functional outcomes do not rely on bone union (and capability of weight‐bearing) as the sole evaluation parameter. Other prerequisites that may affect the situation are the general condition of the patient, pain tolerance, muscle status, physical activities, and psychological status of the patien, which is markedly affected by the prolonged presence of the fixator and its effect on the patient's daily personal, social, and familial activities. All outcome measures in our study improved over time between 6‐months and 2 years. The one‐year mean TOM score is less prone to a ceiling effect (i.e. reaching the upper‐most end on the scale) “activities of daily living” dimension. The average TOM score after 2 years postoperatively only lays at 20% below the upper limit to the scale, while 30% of the patients reached the maximum score of 40 on the TOM scale at a 2‐year follow‐up (Table 1).

According to the modified functional outcome of the Karlstrom– Olerud score, the final functional results were: excellent in 12 cases (48%), good in nine cases (36%), satisfied in two cases (8%), and poor in one case (4%), and these results are fairly reasonable.

The main limitations of our study are a small sample size, the need for a control group to compare the results with other single stages (acute conversion) protocol. Also, the fact that some cases had infected nonunion while others had non‐infected nonunion may have some impact on the outcome.

### *Conclusion*

The technique of two‐stage (delayed conversion) treatment of nonunions of long bone after external fixation is an effective strategy to achieve favorable functional outcomes. It decreases the frame time and incidence of nonunions as well as deformity corrections. For optimization of the functional results, infection must be eradicated well and this should be monitored by clinical and laboratory parameters.

## Declarations

### *Ethics Approval and Consent to Participate*

Ethical approval: This article does not contain any studies with animals.

All procedures performed in our study were in accordance with the ethical standards of the institutional research committee and with the 1964 Helsinki Declaration and its later amendments or comparable ethical standards.

### *Consent for Publication*

Informed consent was obtained from all individual participants included in the study according to the rules of the hospital research ethical committee.

## Availability of Data and Material

The datasets used and analyzed during the current study are available from the corresponding author on request

## Authors' Contributions

Bahaa Ali Kornah: Performed the study design, developed the research question, supervised the surgical technique, and contributed to writing of manuscript.

Mohamed Abdelaziz: Surgical technique, contributed to writing of manuscript, and follow‐up of cases.

Mohamed I Abulsoud: Surgical technique contributed to writing of manuscript, revised the statistical analysis and follow‐up of cases.

Tharwat Abdel Ghani: Surgical technique, contributed to writing of manuscript, and follow up of cases.

Nagi A. Seleem: Surgical technique, contributed to writing of manuscript, and follow up of cases.

Ehab A. Alshal: Surgical technique, contributed to writing of manuscript, and follow‐up of cases.

Mohamed A. Abdel‐AAl: Statistical analysis, preparation of final manuscript, and follow‐up of cases.

## References

[1] Fragomen AT , Rozbruch SR . The mechanics of external fixation. HSS J, 2007, 3: 13–29. 18751766 10.1007/s11420-006-9025-0 PMC2504087

[2] Beltsios M , Savvidou O , Kovanis J , Alexandropoulos P , Papagelopoulos P . External fixation as a primary and definitive treatment for tibial diaphyseal fractures. Strategies Trauma Limb Reconstr, 2009, 4: 81–87. 19714440 10.1007/s11751-009-0062-3 PMC2746273

[3] Einhorn TA . Enhancement of fracture‐healing. JBJS, 1995, 77: 940–956. 10.2106/00004623-199506000-00016 7782368

[4] Papaioannou N , Mastrokalos D , Papagelopoulos PJ , Tyllianakis M , Athanassopoulos J , Nikiforidis PA . Nonunion after primary treatment of tibia fractures with external fixation. Eur J Orthop Surg Traumatol, 2001, 11: 231–235.

[5] Stojković B , Milenković S , Radenković M , Stanojković M , Kostić I . Tibial shaft fractures treated by the external fixation method. Med Biol, 2006, 13: 145–147.

[6] Weber BG , Cech O . Pseudarthrosis: Pathology biomechanics therapie results. Switerland: Hans Huber Medical Publisher, 1976.

[7] Paley D . Problems, obstacles, and complications of limb lengthening by the Ilizarov technique. Clin Orthop Relat Res, 1990, 250: 81‐104. 2403498

[8] Fischgrund J , Paley D , Suter C . Variables affecting time to bone healing during limb lengthening. Clin Orthop Relat Res, 1994, 301: 31–37. 8156692

[9] Maurer DJ , Merkow RL , Gustilo RB . Infection after intramedullary nailing of severe open tibial fractures initially treated with external fixation. J Bone Joint Surg Am, 1989, 71: 835–838. 2745479

[10] Linh HB , Feibel RJ . Tibial lengthening over an intramedullary nail. Tech Orthop, 2009, 24: 279–288.

[11] Sabharwal S , Rozbruch SR . What's new in limb lengthening and deformity correction. JBJS, 2011, 93: 2323–2332. 10.2106/JBJS.K.01215 22258779

[12] Brewster MB , Mauffrey C , Lewis AC , Hull P . Lower limb lengthening: is there a difference in the lengthening index and infection rates of lengthening with external fixators, external fixators with intramedullary nails, or intramedullary nailing alone? A systematic review of the literature. Eur J Orthop Surg Traumatol, 2010, 20: 103–108.

[13] Sun XT , Easwar TR , Manesh S , *et al*. Complications and outcome of tibial lengthening using the Ilizarov method with or without a supplementary intramedullary nail: a case‐matched comparative study. J Bone Joint Surg, 2011, 93: 782–787. 10.1302/0301-620X.93B6.25521 21586777

[14] Monni T , Birkholtz FF , De Lange P , Snyckers CH . Conversion of external fixation to internal fixation in a non‐acute, reconstructive setting: a case series. Strategies Trauma Limb Reconstr, 2013, 8: 25–30. 23475382 10.1007/s11751-013-0157-8 PMC3623921

[15] Apard T , Bigorre N , Cronier P , Duteille F , Bizot P , Massin P . Two‐stage reconstruction of post‐traumatic segmental tibia bone loss with nailing. Orthop Traumatol Surg Res, 2010, 96: 549–553. 20605548 10.1016/j.otsr.2010.02.010

[16] Marín LA , Salido JA , López A , Silva A . Preoperative nutritional evaluation as a prognostic tool for wound healing. Acta Orthop Scand, 2002, 73: 2–5. 11928905 10.1080/000164702317281323

[17] Suk M , Hanson BP , Norvell DC , Helfet DL . Musculoskeletal outcomes measures and instruments. In: AO Handbook, 1st edn. Stuttgart, Germany: Georg Thieme, 2004; 1–444.

[18] Karlström G , Olerud S . Fractures of the tibial shaft a critical evaluation of treatment alternatives. Clin Orthop Relat Res, 1974, 105: 82‐115. 4609658

[19] Van den Bossche MR , Broos PL , Rommens PM . Open fractures of the femoral shaft, treated with osteosynthesis or temporary external fixation. Injury, 1995, 26: 323–325. 7649648 10.1016/0020-1383(95)00050-j

[20] Carmack DB . Conversion from external fixation to definitive fixation: periarticular injuries. J Am Acad Orthop Surg, 2006, 14: S128–S130. 17003184 10.5435/00124635-200600001-00029

[21] Chaudhary MM . Infected nonunion of tibia. Indian J Orthop, 2017, 51: 256–268. 28566776 10.4103/ortho.IJOrtho_199_16 PMC5439310

[22] Brinker MR , O'Connor DP . Outcomes of tibial nonunion in older adults following treatment using the Ilizarov method. J Orthop Trauma, 2007, 21: 634–642. 17921839 10.1097/BOT.0b013e318156c2a2

[23] Emara KM , Allam MF . Ilizarov external fixation and then nailing in management of infected nonunions of the tibial shaft. J Trauma Acute Care Surg, 2008, 65: 685–691. 10.1097/TA.0b013e3181569ecc 18784585

